# Dynamic mRNA and miRNA expression analysis in response to hypoxia and reoxygenation in the blunt snout bream (*Megalobrama amblycephala*)

**DOI:** 10.1038/s41598-017-12537-7

**Published:** 2017-10-09

**Authors:** Shengming Sun, Fujun Xuan, Xianping Ge, Jian Zhu, Wuxiao Zhang

**Affiliations:** 1 Key Laboratory of Genetic Breeding and Aquaculture Biology of Freshwater Fishes, Ministry of Agriculture, Freshwater Fisheries Research Centre, Chinese Academy of Fishery Sciences, Wuxi, 214081 P.R. China; 2 Jiangsu Provincial Key Laboratory of Coastal Wetland Bioresources and Environmental Protection, Yancheng City, Jiangsu Province 224002 P.R. China; 3 Wuxi Fisheries College, Nanjing Agricultural University, Wuxi, 214081 P.R. China

## Abstract

Adaptation to hypoxia is a complex process involving various pathways and regulation mechanisms. A better understanding of the genetic influence on these mechanisms could permit selection for hypoxia-sensitive fish. To aid this understanding, an integrated analysis of miRNA and mRNA expression was performed in *Megalobrama amblycephala* under four acute hypoxia and reoxygenation stages. A number of significantly differentially-expressed miRNAs and genes associated with oxidative stress were identified, and their functional characteristics were revealed by GO function and KEGG pathway analysis. They were found to be involved in HIF-1 pathways known to affect energy metabolism and apoptosis. MiRNA-mRNA interaction pairs were detected from comparison of expression between the four different stages. The function annotation results also showed that many miRNA-mRNA interaction pairs were likely to be involved in regulating hypoxia stress. As a unique resource for gene expression and regulation during hypoxia and reoxygenation, this study could provide a starting point for further studies to better understand the genetic background of hypoxia stress.

## Introduction

Hypoxia in fish can lead to adverse effects on growth, behaviour, reproduction and overall survival^1–6^. Hypoxia occurs in aquatic environments when the concentration of dissolved oxygen is low because the rate of consumption by living organisms is greater than the supply^7^. Rapid and sometimes damaging changes in oxygen level are common in the densely-stocked, shallow ponds common to the aquaculture industry. One major reason is the high nutrient input, which often triggers phytoplankton blooms; these in turn cause a marked decrease in dissolved oxygen levels during the night when photosynthesis ceases. Other factors affecting water oxygen content include salinity, rate of photosynthesis, presence of pollution, wind speed, temperature, hour of the day and season^8^. The survival of the fish depends on their ability to adapt rapidly to the changing levels of oxygen via specialized anatomic, behavioural and physiological strategies^9^.

Under the condition of hypoxia, or hypoxia followed by reoxygenation, oxygen reacts with various chemical species including accumulated electrons via the one-electron mechanism in mitochondria, and also with accumulated degradation products such as hypoxanthine and xanthine. This ultimately results in a rise in production of reactive oxygen species (ROS)^10–12^.

The mechanisms of hypoxia and reoxygenation are of particular interest when the fish concerned have a high economic value. The blunt snout bream, also known as the Wuchang bream (*Megalobrama amblycephala*), is a herbivorous freshwater fish that is farmed commercially in China. It is distributed in the affiliated lakes of the Yangtze River, such as Liangzi Lake, Poyang Lake and Yuni Lake^13–15^. Although it has long been recognised as one of the most important freshwater species^16^, it is not suitable for high-density pond farming because of its relatively low hypoxia threshold. Its tolerance is low compared to fish from the same family, such as the common carp *Cyprinus carpio* ^17^. Its sensitivity to hypoxia is such that even a short period (<2 h) of hypoxia (<0.5 mg/L) at room temperature can be lethal^18^. Although this sensitivity to hypoxia has implications for the welfare and culture of *M. amblycephala*, it could allow this species to be used as a model organism to explore the molecular mechanisms of acute hypoxia and reoxygenation via the transcriptome.

Next-generation sequencing (NGS)-based RNA sequencing (RNA-seq) methods of transcriptome analysis not only allow sequences to be acquired for gene discovery but also help to identify which transcripts are involved in hypoxic stress^19–21^. Recent advances in RNA-seq have generated an unprecedented global view of the transcriptome and have also provided a more efficient method to explore the transcriptional landscape^22,23^. MicroRNA (miRNA) is one of the epigenetic mechanisms which can regulate gene expression in a tissue-specific manner^24^. MiRNAs have been identified in a wide variety of organisms and are currently being investigated in laboratory fish models such as the zebrafish *Danio rerio* ^25^, the Japanese medaka *Oryzias latipes* ^26^ and marine medaka *Oryzias melastigma* ^27^. Integrated analysis of mRNA and miRNA expression profiles has been performed in the zebrafish for optic nerve regeneration, in *M. amblycephala* in response to intermuscular bone development and in darkbarbel catfish (*Pelteobagrus vachelli*) in response to hypoxia^28–30^. A possible next step for research could therefore be to investigate whether hypoxia-induced dysregulation of miRNA causes oxidative tress impairment, and if so to identify the mechanism. A potentially more reliable method than *in* vivo trials for predicting miRNA-mRNA interactions is to integrate real mRNA and miRNA transcriptomic data into *in silico* target predictions^31^.

We therefore used NGS to determine mRNA-seq and miRNA-seq in the livers of both control and hypoxia-treated *M. amblycephala*, to investigate the molecular mechanisms of hypoxia adaptation. We identified hypoxia time specific expressed mRNAs and miRNAs to further understand the molecular mechanisms of hypoxia adaptation. The study also found various interaction networks and regulatory modes of mRNAs and miRNAs, based on the integrated analysis of miRNA and mRNA expression profiles. Finding the molecular mechanisms of hypoxia adaptation in fish will not only help us to understand the evolution of the hypoxia-signalling pathway but will also inform breeding of hypoxia-tolerant fish strains.

## Results and Discussion

### Assembly and annotation of reference transcriptome

To obtain a reference transcriptome for *M. amblycephala* in response to hypoxia and reoxygenation, a RNA-seq library was constructed using RNA from samples of four stages. A total of 425,901,164 raw reads were generated using high-throughput sequencing. After quality control, approximately 416,787,182 high-quality reads with a Q20 percentage of 96.51% and GC percentage of 46.08% were available for analysis (Table 1). A total of 293,928 contigs were generated using the Trinity de novo assembler, with an average length of 566 bp and an N50 of 667 bp. A total of 279,747 unigenes were generated with an average length of 534 bp (Table 1). The length distribution of these contigs is shown in Supplementary Figure S1. A total of 89,118 unigenes were successfully annotated via alignment to reference databases. The NR, Swiss-Prot, KEGG and KOG databases were used to annotate 70,747 (79.39%), 58,498 (65.64%), 55,606 (62.40%) and 40,166 (45.07%) unigenes, respectively (Table 1). Annotation was based on the NCBI NR database, E-value distribution, similarity distribution and species distribution of the result of NR annotation (Supplementary Figure S2). All the data are available at the NCBI SRA database (SRP100308).

**Table 1:** Assembly and annotation results of transcriptome sequencing.

Category	Value	Database	Number of unigenes
Total raw reads	425,901,164	NR	70,747
Total clean reads	416,787,182	SwissProt	58,498
Average Q20 percentage	96.51%	KOG	40,166
Average GC percentage	46.08%	GO	18,723
Total number of contigs	293,928	KEGG	55,606
Total length (bp) of contigs	166,386,170	Total number of annotated unigenes	89,118
Mean length (bp) of contigs	566	None annotated unigenes	190,629
N50 of contigs	667		
Total number of unigenes	279,747		
Total length (bp) of unigenes	149,429,722		
Mean length (bp) of unigenes	534		

**Figure 1:**
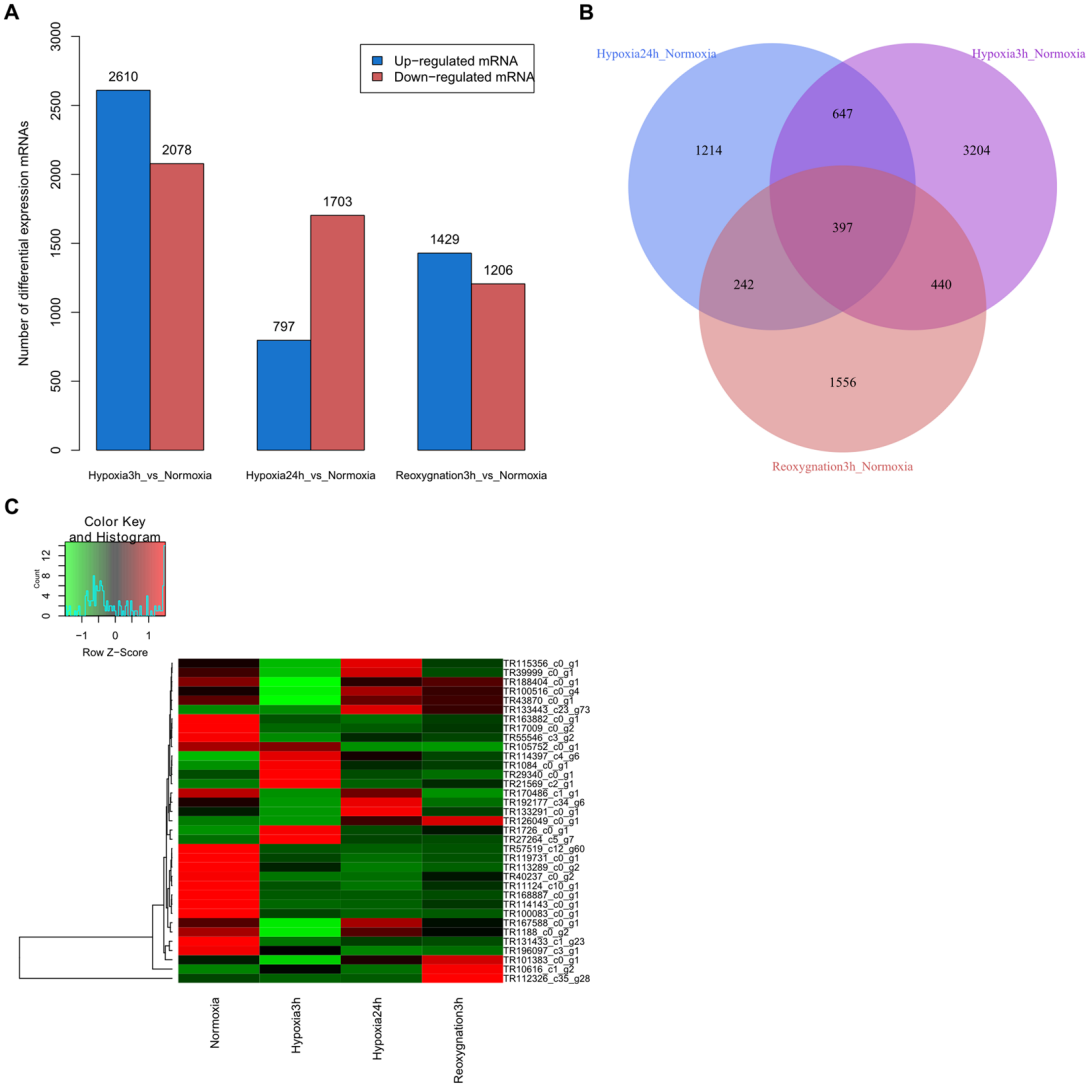
Differentially-expressed mRNA profiles. Notes: (**A**) The number of up/down-regulated DEGs in each comparison analysis group; (**B**) Venn diagram of differential expression between adjacent/nonadjacent pairwise comparisons; (**C**) Hierarchical cluster analysis of significantly decreased/increased expression in mRNA.

To identify the functional genes of *M. amblycephala* in response to hypoxia and reoxygenation, 12 gene expression profiling libraries were generated from the four stages, with three biological replicates. The mapping results for the 12 libraries are shown in the Table 2, and the distributions of unique reads were compared to indicate gene coverage. Similar coverage was obtained for all 12 libraries. Principal component analysis showed that the biological replicates had very similar expression levels, suggesting good reproducibility of the method (Supplementary Figure S3). A total of 7,756 differential expressed genes (DEGs) were found between hypoxia 3 h, hypoxia 24 h, reoxygenation 3 h and normoxia conditions. Of these DEGs, 4658, 2500 and 2635 genes were expressed in response to hypoxia 3 h, hypoxia 24 h and reoxygenation 3 h, respectively (Fig. 1A). The Venn diagram (Fig. 1B) of the DEGs shows that most genes were not expressed in all hypoxia stages. This suggests that the mechanisms and pathways involved in response to hypoxia are different during hypoxia and post-hypoxia recovery (Fig. 1B). Hierarchical clustering of partial differentially-expressed mRNAs was seen between different stages (Fig. 1C). Annotation revealed hundreds of hypoxia-related genes in the transcriptome (Supplementary Table S1), such as hypoxia inducible factors (HIF-1 and HIF-2), heat shock proteins (HSP70 and HSP90) and enzymes involved in glycolysis (hexokinase, phosphofructokinase, pyruvate kinase).

**Table 2:** Alignment results of 12 libraries mapped to the reference transcriptome.

Sample name	Total reads	Total base pairs	Total mapped reads	Unique match	Multi- position match	Total unmapped reads
Hypoxia 24 h-1	35,332,588 (100.00%)	5,299,888,200 (100.00%)	27,936,572 (79.07%)	13,131,576 (74.33%)	836,710 (4.74%)	7,396,016 (20.93%)
Hypoxia 24 h-2	21,959,482 (100.00%)	3,293,922,300 (100.00%)	17,363,868 (79.07%)	8,061,503 (73.42%)	620,431 (5.65%)	4,595,614 (20.93%)
Hypoxia 24 h-3	35,720,160 (100.00%)	5,358,024,000 (100.00%)	29,051,252 (81.33%)	13,458,989 (75.36%)	1,066,637 (5.97%)	6,668,908 (18.67%)
Hypoxia 3 h-1	39,100,038 (100.00%)	5,865,005,700 (100.00%)	30,924,992 (79.09%)	14,034,653 (71.79%)	1,427,843 (7.30%)	8,175,046 (20.91%)
Hypoxia 3 h-2	37,023,622 (100.00%)	5,553,543,300 (100.00%)	30,154,654 (81.45%)	14,010,083 (75.68%)	1,067,244 (5.77%)	6,868,968 (18.55%)
Hypoxia 3 h-3	36,486,806 (100.00%)	5,473,020,900 (100.00%)	28,880,240 (79.15%)	13,280,302 (72.80%)	1,159,818 (6.36%)	7,606,566 (20.85%)
Normoxia-1	38,366,026 (100.00%)	5,754,903,900 (100.00%)	30,185,960 (78.68%)	14,006,684 (73.02%)	1,086,296 (5.66%)	8,180,066 (21.32%)
Normoxia-2	34,179,730 (100.00%)	5,126,959,500 (100.00%)	27,718,630 (81.1%)	12,913,227 (75.56%)	946,088 (5.54%)	6,461,100 (18.90%)
Normoxia-3	34366628 (100.00%)	5,154,994,200 (100.00%)	28,410,456 (82.67%)	13,310,863 (77.46%)	894,365 (5.20%)	5,956,172 (17.33%)
Reoxygnation 3 h-1	36092204 (100.00%)	5,413,830,600 (100.00%)	29,316,068 (81.23%)	13,686,571 (75.84%)	971,463 (5.38%)	6,776,136 (18.77%)
Reoxygnation 3 h-2	35452202 (100.00%)	5,317,830,300 (100.00%)	29,455,916 (83.09%)	13,865,677 (78.22%)	862,281 (4.86%)	5,996,286 (16.91%)
Reoxygnation 3 h-3	32707696 (100.00%)	4906154400 (100.00%)	26610000 (81.36%)	12354083 (75.54%)	950917 (5.81%)	6097696 (18.64%)

### Functional analysis of DEGs during hypoxia and reoxygenation

The liver in fish has relatively high metabolic oxygen demands^32^. Previous studies of expression profiling in fish have found that a cluster of genes involved in oxygen utilization are differentially expressed in response to hypoxia^33–36^. An increased hemoglobin mRNA expression levels has been found during hypoxia acclimation in liver of *M. amblycephala*, which was similar with previous study in muscle of this specie^37^. It was beneficial for utilization of circulating oxygen during hypoxia as that could facilitate the diffusion of oxygen.

The electron transport chain in mitochondria is a major site of ROS production. Hypoxic stress partially inhibits mitochondrial electron transport, leading to redox changes in the electron carriers and increasing ROS production at complex III^38,39^. A compromised antioxidant defence or inhibition of electron flow can disrupt the delicate balance between antioxidant defence and ROS production^40^, thus leaving *M. amblycephala* susceptible to ROS damage during hypoxia. Interestingly, superoxide dismutase (SOD), catalase (CAT) and nuclear factor erythroid 2-related factor 2 (Nrf2) was significantly higher in *M. amblycephala* in response to reoxygenation 3 h, previous study have confrimed that the Nrf2 in modulating the activation of antioxidant defences at both transcriptional and functional levels under reoxygenation in *Rhamdia quelen* ^41^, *Cyprinus carpio* ^42^ and *Pelteobagrus vachelli* ^43^. Although the antioxidant enzyme levels could only reflect the antioxidant capacity rather than the status of oxidative stress, previous study confrimed that ROS still significant increased in *M. amblycephala* during the recovery from severe acute hypoxia (DO: 1.0 ± 0.2 mg/L) treatment^44^, which supports the hypothesis that anticipatory preparation takes place in order to deal with the encountered oxidative stress during the recovery from hypoxia as proposed by M. Hermes-Lima.

Enrichment analysis is an effective way to identify those KEGG pathways which frequently occur in a tissue, against a background of the whole body transcriptome^45,46^. A total of 532 DEGs were mapped into 108 KEGG pathways in response to hypoxia and reoxygenation (Supplementary Table S2). By performing the KEGG pathway analyses, top 20 significantly changed pathways were identified in response to hypoxia and reoxygenation, respectively (Figure S4). Among these top 20 pathways, “Signal transduction” were the most commonly represented subclasses in hypoxia 3 h vs. normoxia group. “HIF-1 signaling pathway”, “Insulin signaling pathway”, “AMPK signaling pathway”, “P53 signaling pathway” and “FoxO signaling pathway” are included in the “Signal transduction” subclass, suggesting that a number of other transcription factors are activated either directly or indirectly in response to acute hypoxia. “Immune system” were the most commonly represented subclasses in hypoxia 24 h vs. normoxia group. “Viral myocarditis”, “Viral carcinogenesis”, “Phagosome”, “Complement and coagulation cascades” are included in the “Immune system” subclass. The present results imply that our prolonged hypoxia treatment affected immunity-related genes involved in these pathways in *M. amblycephala*. “Glycolysis/Gluconeogenesis”, “Fructose and mannose metabolism”, “Galactose metabolism”, “Pentose phosphate pathway”, “Starch and sucrose metabolism”, “Fatty acid metabolism” and “Biosynthesis of amino acids” have also been significantly enriched in reoxygenation 3 h vs. normoxia group. The enrichment of “Substance metabolism” subclasses may be necessary to cope with the energy demand balance from the liver of *M. amblycephala* in respond to reoxygenation 3 h.

While the hypoxia-inducible factor (HIF-1) plays a major role in controlling the ubiquitous transcriptional response to hypoxia, we focused our analyses on genes likely to be relevant to hypoxia 3 h and found that the HIF-1 signalling pathway co-operated with the AMP-activated protein kinase (AMPK) pathway to regulate and control hypoxia stress (Supplementary Figure S5). HIF-1α is rapidly broken down by prolyl hydroxylases in normoxia, but is stabilised in hypoxia^47–50^. This suggests a model of hypoxia-induced regulation of the HIF-1α transcript in fish, which could be similar to typical HIF-1α transcriptional regulation in mammals^51^. In the present study, AMPK was significantly up-regulated, consistent with previous studies in crucian carp *Carassius auratus* ^52^ and gibel carp *Carassius carassius* ^53^. AMPK is activated by increases in the cellular AMP:ATP ratio caused by hypoxia stresses that either reduce ATP production or ATP-producing catabolic pathways such as fatty acid oxidation and glycolysis^54,55^, suggesting that anaerobic metabolism has probably taken the place of aerobic metabolism during hypoxia 3 h since *M. amblycephala* suffocation point ranges from 0.64 to 0.35 mg/L^56^. Aerobic metabolism is less efficient than aerobic respiration when producing energy, and a considerable increase in glycolytic flux is needed to avoid a detrimental decrease of cellular ATP^57–59^. The KEGG analysis revealed that some pathways related to immune response were significantly down-regulated in response hypoxia 24 h, it was suggested that hypoxia stress can modulate and compromise the innate immune response as previous shown in *M. amblycephala* ^60^. It is noteworthy that “Oxidative phosphorylation” was found to be the most frequently represented subclasses in *M. amblycephala* in response to reoxygenation 3 h, suggesting increase total cellular ATP content by induced glycolysis and mitochondria oxidative phosphorylation in fish for reoxygenation. In addition, similar with previous study in *M. amblycephala* ^44^, some important immunity-related pathway have also been significantly enriched, suggesting that the difference of energy metabolism pattern and immune response is the main biological process of hypoxia and recover.

### Sequence and expression profiling of hypoxia-related microRNAs

**Figure 2:**
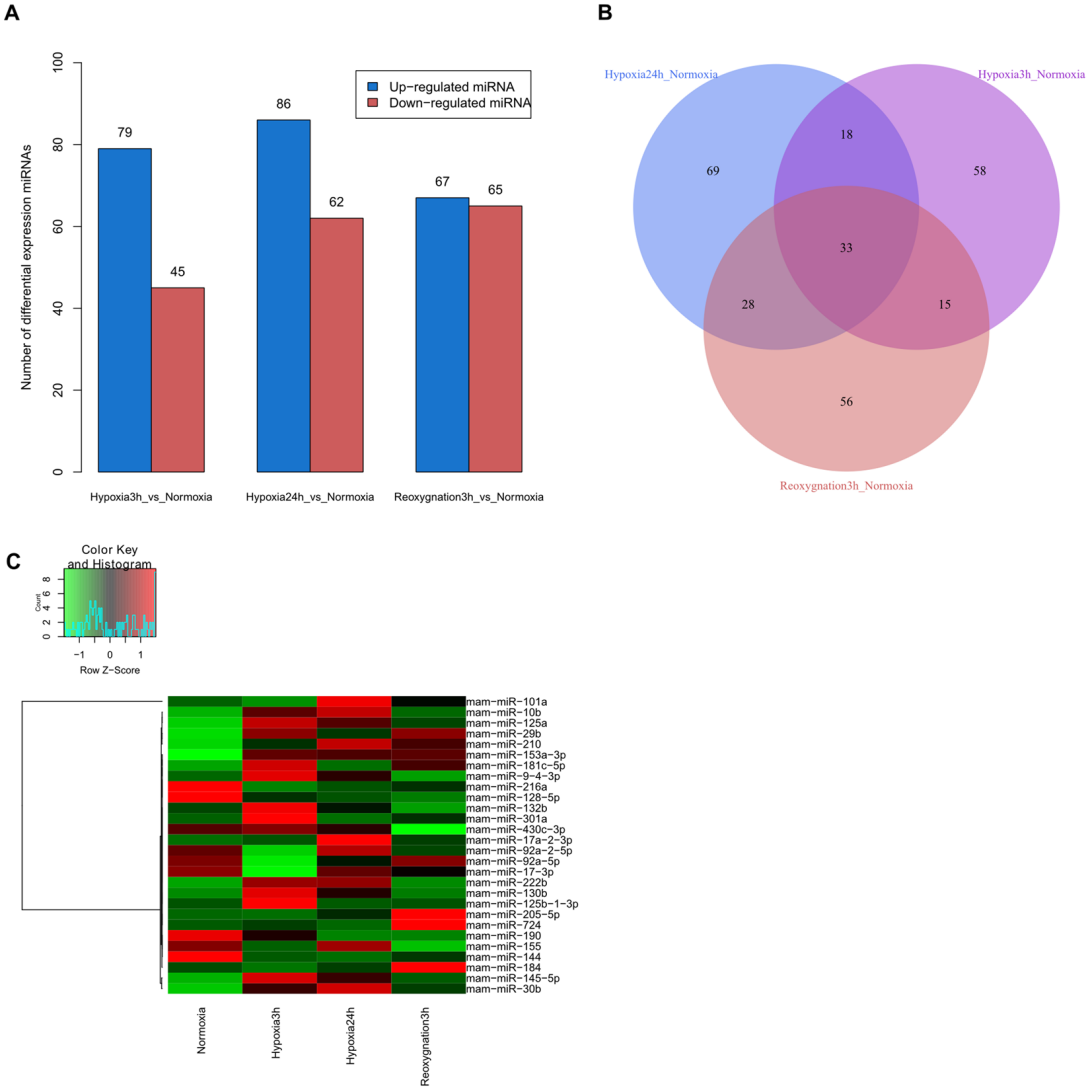
Differentially-expressed miRNAs profiles. Note: (**A**) The number of up/down-regulated miRNAs in each comparison analysis group; (**B**) Venn diagram of differential expression between adjacent/nonadjacent pairwise comparisons; (**C**) Hierarchical cluster analysis of significantly decreased/increased expression in miRNAs.

The four libraries of normoxia, hypoxia 3 h, hypoxia 24 h and reoxygnation 3 h yielded a total of 11,462,022, 11,296,460, 11,076,614 and 11,224,233 raw reads, respectively. After discarding low-quality reads, 3′ and 5′ adaptors and sequences with <18 nt, the numbers of clean small RNA reads were 10,428,292, 8,925,217, 8,894,813 and 9,025,112, respectively (Supplementary Table S3-1). Most of the small RNAs were 21–23 nt in length, with 22 nt being the most abundant length (>75%). The analysis of common and specific sequences between libraries is shown in Supplementary Table S3-2. After NCBI Genbank and Rfam database alignment, rRNA, tRNA, snRNA and snoRNA were annotated and removed (Supplementary Table S3-3). To evaluate miRNA activities across the four different stages of hypoxia and reoxygnation, we performed a time course analysis of differential miRNA expression by comparing control and treatment stages (Supplementary Table S4). Using a |log_2_FC| of ≥1 and reads ≥100 as the cut-off, we identified the following differentially-expressed miRNAs in adjacent pairwise comparisons (Fig. 2A): in normoxia-vs-hypoxia 3 h there were 132 (40 up-regulated and 92 down-regulated); in normoxia-vs-hypoxia 24 h there were 120 (69 up-regulated and 51 down-regulated); and in normoxia-vs-reoxygnation 3 h there were 174 (108 up-regulated and 66 down-regulated) miRNAs.

Through Venn diagram analysis, 33 overlapping differentially-expressed miRNAs were identified in the three pairwise and three nonadjacent pairwise comparisons (Fig. 2B). Hierarchical clustering of partial differentially-expressed miRNAs was seen between different stages (Fig. 2C).

**Figure 3:**
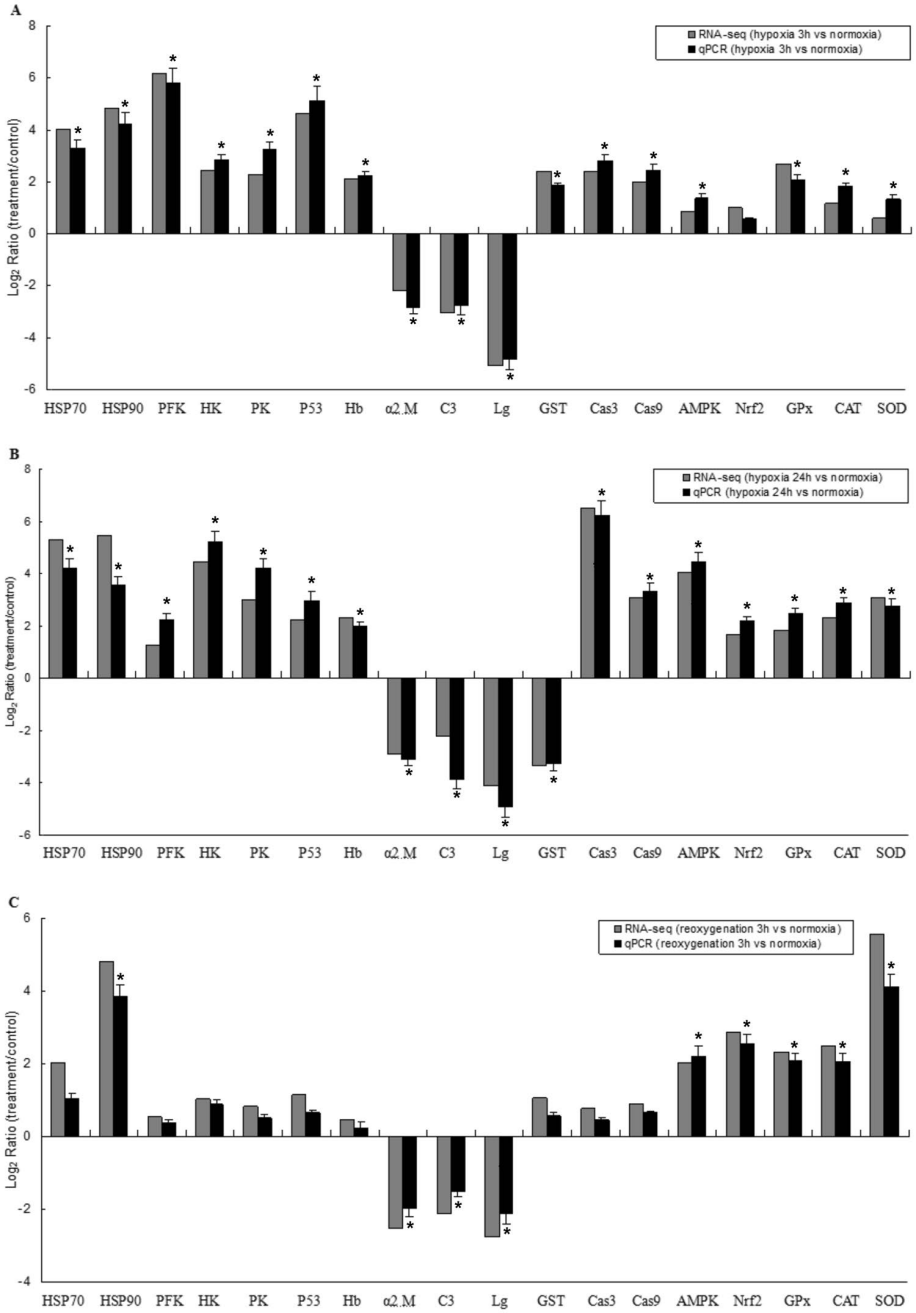
Comparison of expression levels for the 18 significantly-expressed mRNAs using RNA-Seq and qRT-PCR. Note: Statistical analysis of differences was conducted by one-way analysis of variance (ANOVA) using SPSS 19.0 software. Error bars represent standard deviation of three replicates. The asterisks above the bars represented statistically significant differences from hypoxia 3 h vs. normoxia group (**A**), hypoxia 24 h vs. normoxia group (**B**), and reoxygenation 3 h vs. normoxia group (**C**), respectively (*P < 0.05).

In this study, we found several miRNAs that were significantly differentially expressed in response to hypoxia. For example, miR-9 and miR-124 are both known to play a crucial role in neurogenesis and neuronal development, particularly in the regulation of neural differentiation, proliferation and cell migration^61–63^. Both miR-143 and miR-101 can directly target the key glycolytic enzyme hexokinase under hypoxic conditions in tissue cells^64,65^, as we known, hexokinase is the first key regulatory enzyme of the glycolytic pathway^66^, previous study show that hexokinase could accelerates the rate of glycolysis to produce ATP anaerobically in *Gadus morhua* in response to hypoxia stress^36^, which was consitent with our results of this study in *M. amblycephala* (Fig. 3). In addition, miRNAs implicated in the regulation of apoptosis include miR-210, miR-21, miR-17 and miR-29, which were found to be expressed in the current study. Of these, miR-210 and miR-21 exert broad pleiotropic effects by targeting genes involved in mitochondrial function, apoptosis, cell cycle arrest and cell survival^67,68^, while miR-29 is thought to be pro-apoptotic, suppressing tumour growth in mice^69,70^. Those miRNA above were predicted target gene including p53, caspase-3 and caspase-9, were significant increased in liver of *M. amblycephala* in response to hypoxia (Fig. 3), The tumor suppressor protein p53 is a universal sensor of environmental stress, it plays a critical role in promoting the survival or death of cells exposed to agents that cause DNA damage^71^, caspase 3 and caspase-9 play a pivotal role in mitochondria apoptosis pathway^72^, suggesting apoptosis pathway in *M. amblycephala* as a hypoxic sensitive fish was induced by hypoxia stress. Therefore, some of the miRNAs that are differentially expressed between hypoxia and normoxia stages are likely to play important roles in the physiological response.

### Quantitative analysis of miRNA and mRNA expression

**Figure 4:**
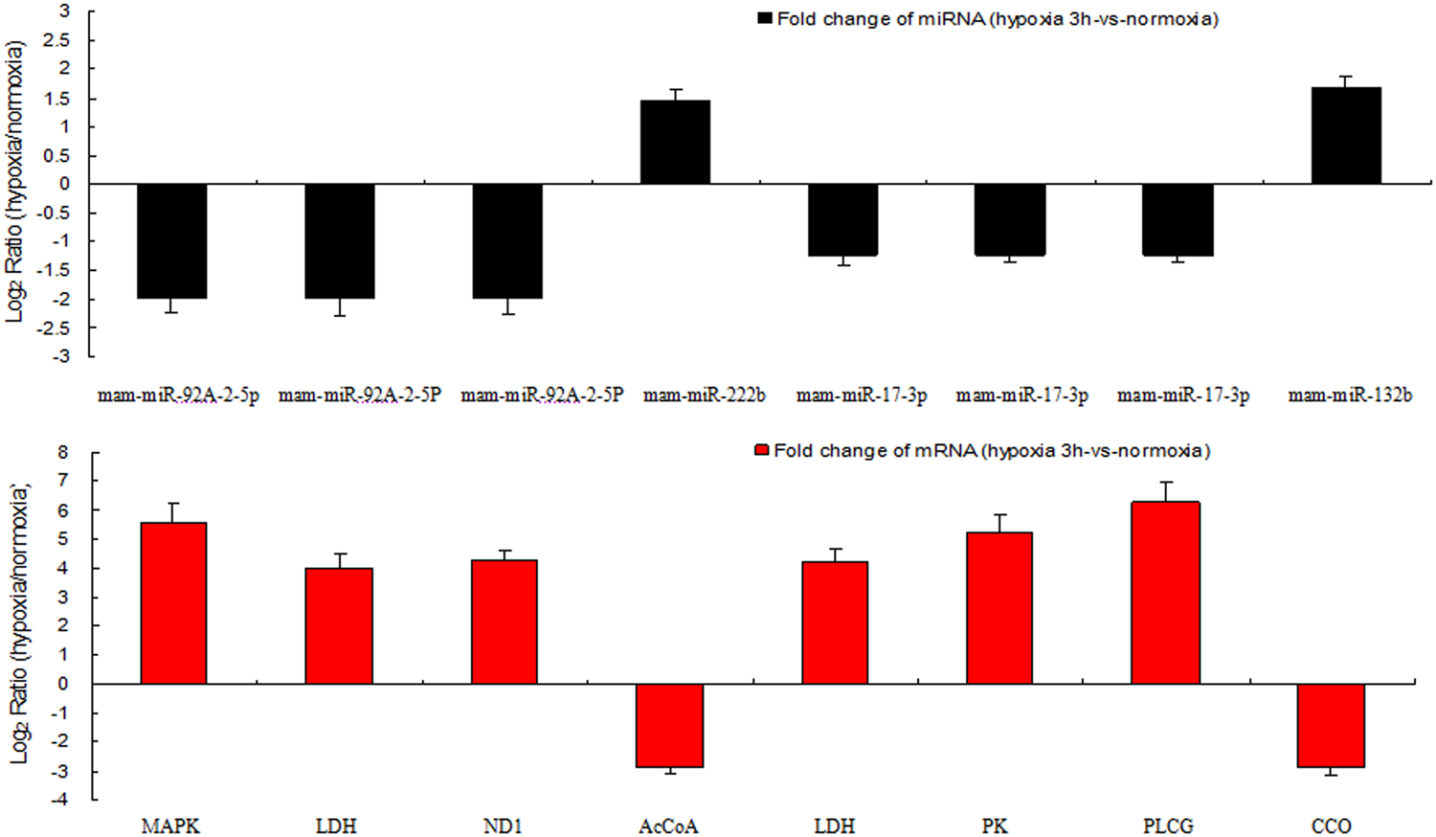
Comparison of expression patterns of miRNA-mRNA interaction pairs using qRT-PCR under hypoxia 3 h.

To validate the RNA-seq results, 18 genes that showed a high level expression (or are known to be important in the stress response) were selected for qRT-PCR analysis. Same expression trends were found between qRT-PCR and the Illumina data (Fig. 3). Eight mRNAs were manually selected as representatives for their potential roles in hypoxia response according to their annotations and their potential relationship with four hypoxia-responsive miRNAs. These genes encode AMP-activated protein kinase (miR-92a-2-5p-AMPK), lactate dehydrogenase (miR-92a-2-5p-LDH), NADH dehydrogenase subunit 1 (miR-92a-2-5p-ND1), acetyl-coenzyme A synthetase (miR-222b-AcCoA), lactate dehydrogenase (miR-17-3p-LDH), pyruvate kinase (miR-17-3p-PK), 1-phosphatidylinositol 4,5-bisphosphate phosphodiesterase (miR-17-3p- PLCG), cytochrome coxidase subunit (miR-132b-CCO). A similar result was seen in the validation of the miRNA-mRNA interaction pairs: four interaction pairs had the same expression pattern as the sequencing data (Fig. 4). We believe the results confirm the credibility of the molecular resources and sequencing data identified in our study.

**Figure 5:**
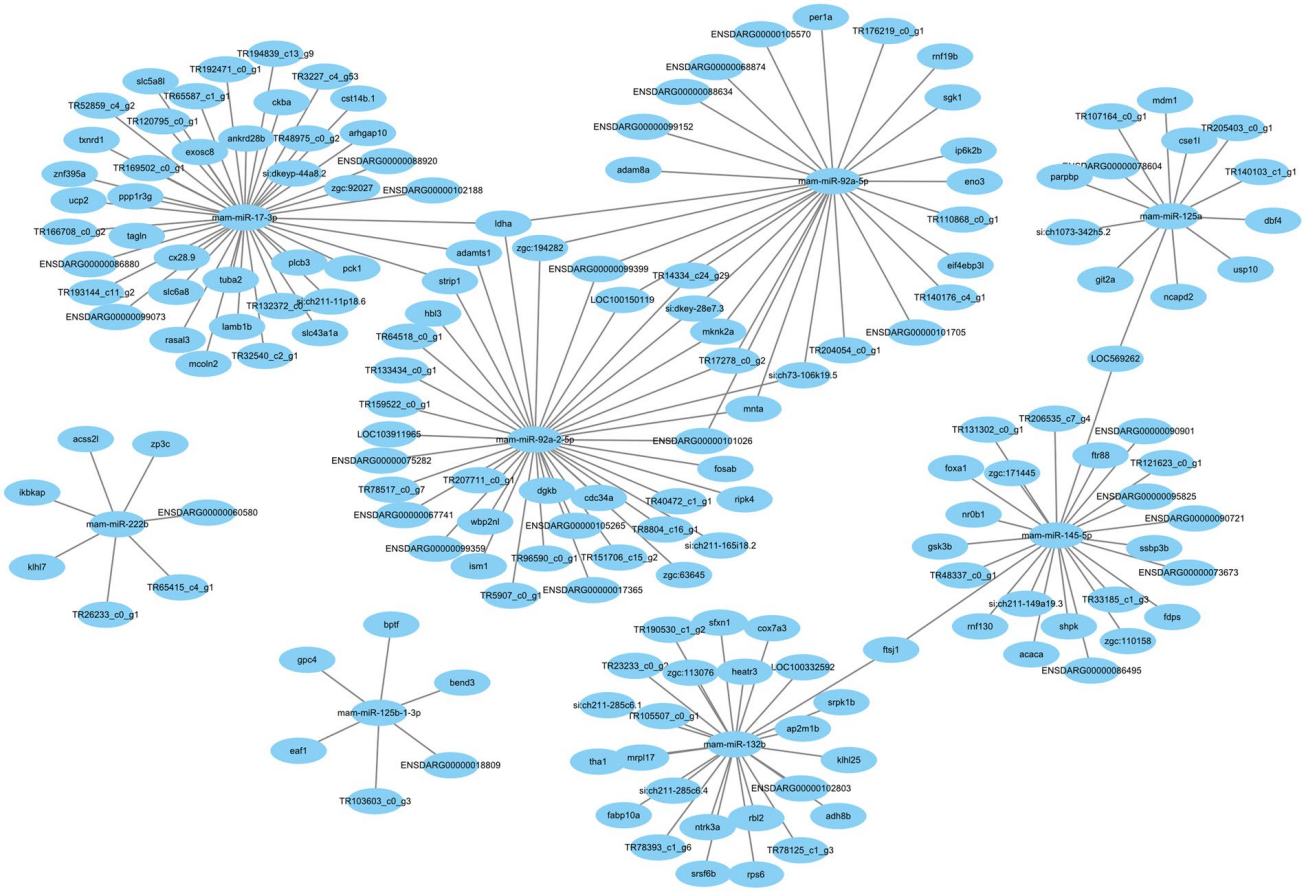
MiRNA-mRNA interaction diagrams under hypoxia 3 h.

The complexity of the miRNA-mRNA interaction pairs involved in specific biological processes has been an exciting and challenging field of study^73^, Due to the limited genetic background of *M. amblycephala* and that the present ESTs may be the noncoding RNA, our study describes here the interaction between 65 genes with annotation and 8 miRNAs, those miRNA-mRNA interaction network during early hypoxia 3 h help us understand the roles for specific miRNAs or miRNA-mRNA interactions (Fig. 5).

## Conclusion

This study is the first integrated analysis of miRNA and mRNA expression during hypoxia and reoxygenation in a fish species. Putative miRNAs and genes associated with hypoxia were identified in the study. We found the obvious negative correlation between miRNA and their target mRNAs, providing several miRNA-mRNA interaction networks in *M. amblycephala* in response to hypoxia. This study generated fundamental molecular resources across four hypoxia and reoxygenation stages, which can be used to further understand the molecular processes of hypoxia stress. Fouther analysis of miRNA-mRNA regulatory network from other tissues or cell types by bioinformatics analysis will be required.

## Materials and Methods

### Ethics statement

All animals and experiments were conducted in accordance with the “Guidelines for Experimental Animals” of the Ministry of Science and Technology (Beijing, China). All efforts were made to minimize suffering. All experimental procedures involving fish were approved by the institution animal care and use committee of the Chinese Academy of Fishery Sciences.

### Sample collection

Two hundred of healthy *M. amblycephala* juveniles with a mean weight of 50.56 ± 5.15 g were obtained from the Freshwater Fisheries Research Center, Chinese Academy of Fishery Sciences, China. The fish were immediately transferred to the aquatic laboratory and kept in six 500-L fiberglass tanks (n = 30 fish/tank). During acclimation, each tank was filled with pre-aerated municipal water kept under the following conditions: 24.6 ± 0.5 °C, pH 8.2 ± 0.08, dissolved oxygen 6.5 ± 0.2 mg/L, total ammonia-nitrogen 0.08-0.09 mg/L, with natural photoperiod. The control group was maintained under normoxic conditions (6.5 ± 0.2 mg/L). In the treatment tanks, hypoxic conditions (1.5 ± 0.1 mg/L dissolved oxygen) were maintained by nitrogen manipulation^74^. Fish were randomly allocated to one of four treatment groups: hypoxia for 0 h (normoxia, control group), hypoxia for 3 h, hypoxia for 24 h and hypoxia for 24 h + 3 h recovery in normoxia. Liver samples were obtained by dissection after mild anaesthetisation in a eugenol bath (1:10,000), and immediately frozen in liquid nitrogen and stored at −80 °C until processing.

### RNA isolation, cDNA library construction and sequencing

Total RNA was extracted from the tissue using TRIzol reagent (Invitrogen, Carlsbad, CA, USA) according to the manufacturer’s directions. RNA samples were digested using DNase I to remove potential genomic DNA. The integrity and size distribution were assessed using a Bioanalyzer 2100 with RNA 6000 Nano Labchips (Agilent technologies, Santa Clara, CA, USA), and all samples were standardised to 500 ng/μl. Three sequencing libraries and one miRNA transcriptome library were constructed for each hypoxia and reoxygenation condition. Next, 12 sequencing libraries were constructed using a TruSeq™ RNA Sample Preparation Kit (Illumina) according to the manufacturer’s directions. The next step was KAPA quantification and dilution, after which the library was sequenced on an Illumina HiSeq. 2500 with 125-bp paired-end reads. After removing adaptor sequences, ambiguous N nucleotides (those an N ratio <5%) and low quality sequences (reads with <50% bases of quality value), the remaining clean reads were assembled using Trinity software^75^. This was performed as described for a *de novo* transcriptome assembly without a reference genome. The process generated the reference sequences, including several EST sequences available in the public domain for comparative transcriptome study. All data were made available in the NCBI SRA database (https://www.ncbi.nlm.nih.gov/sra/?term=SRP100308).

### Functional annotation of assembled contigs

Functional annotation of the assembled reference sequences was performed by homology searches against the NCBI non-redundant (NR) protein database^76^, The Universal Protein Resource (UniProt-SwissProt) database^77^ and the Kyoto Encyclopaedia of Genes and Genomes (KEGG) database^78,79^. The assembled transcriptome contigs were subjected to similarity searches against the NCBI NR protein database using BLASTx with an e-value cut-off of 1e-10. A gene name and description was assigned to each contig based on the BLASTx hit with the highest score.

### Differential gene expression analysis

The cleaned reads were mapped to the assembled reference transcriptome using Bowtie^80^, and about 70% of the reads for each sample could be mapped to the reference (Table 1). Gene and isoform abundances were quantified using RSEM according to the Trinity-assembled transcriptome. Gene expression was measured as fragments per kilobase of exon per million reads mapped (FPKM). Finally, we normalised the expression levels in each sample using edgeR and obtained the differentially-expressed transcripts using pairwise comparisons^81^. The following threshold values were used to judge the significance of DEGs: a |log_2_(fold change)| of ≥1 and a false discovery rate of ≤0.05. GO analysis was conducted on the assembled transcriptome using InterProScan (http://www.ebi.ac.uk/Tools/pfa/iprscan/) and integrated protein databases with default parameters. The GO terms associated with transcriptome contigs were then obtained for describing their biological processes, molecular functions and cellular components^32^. For pathway enrichment analysis, all DEGs were mapped to terms in the KEGG database and searched for significantly enriched KEGG terms compared to the whole transcriptome background^32^. GO and KEGG were performed using the ultrageometric test to identify which DEGs were significantly enriched in GO terms (P-value ≤ 0.05) and KEGG pathways (q-value ≤ 0.05) compared with the whole transcriptome background.

### MiRNA sequencing and differential expression analysis

Four small RNA libraries were constructed and sequenced as previously described^82^. The Rfam database (ftp://ftp.sanger.ac.uk/pub/databases/Rfam/9.1/) database and the GenBank noncoding RNA database (http://blast.ncbi.nlm.nih.gov/) were searched using BLAST for the high-quality reads obtained. Once the rRNA, tRNA, snRNA and other ncRNA sequences were annotated, they were then aligned to mRNA exons and introns to screen and remove degraded fragments. We also mapped selected sequences to the reference transcriptome (with a tolerance of one mismatch in the seed sequence) to analyse their expression and distribution on the genome. To determine novelty and species specificity, the remaining clean reads were aligned against known deuterostome pre-miRNAs in miRbase 20.0.

Differences in miRNA expression between the hypoxia and reoxygenation stages were determined as |log_2_(fold change)| >1, and significance was set at a P-value of ≤0.05. Subsequently, differences in gene expression which were complementary with corresponding miRNA expression values were selected and analysed with both Targetscan (http://www.targetscan.org/) and MiRanda (http://www.microrna.org) to predict the miRNA target^83^. Finally, miRNA-mRNA pairs were detected and their interaction analysed based on the target prediction, function annotation and negative regulation mechanism of mRNA and miRNA. The resulting miRNA-mRNA interaction networks were displayed using visualising maps.

### Quantitative PCR for miRNA and mRNA expression

The sequencing data were validated as follows: 18 mRNAs which showed significant increases or decreases in expression among “hypoxia 3 h vs normoxia”, “hypoxia 24 h vs normoxia” and “reoxygenation 3 h vs normoxia” stage were identified, along with four miRNA-mRNA interaction pairs from the paired comparison of normoxia and hypoxia for 3 h. Specific qRT-PCR primers and microRNA stem-loop RT were used in this study (Supplementary Table S6). High quality total RNA, miRNAs and mRNAs were obtained and then reverse transcribed using a PrimeScript RT reagent kit with gDNA Eraser (TaKaRa, Japan). Quantitative real-time PCR (RT qPCR) analyses of the miRNAs and mRNAs were performed using SYBR Green PCR Master Mix (TaKaRa) according to the manufacturer’s directions. The internal RT qPCR controls for the miRNA and mRNA were *M. amblycephala* U6 snRNA and β-actin, respectively. All PCRs were performed in triplicate. Relative expression levels were measured in terms of threshold cycle value (Ct) and were normalised using the equation 2^−ΔΔCt^ ^84^.

## Electronic supplementary material

Additional figures

Dataset 1

## References

[1] Pollock MS (2007). The effects of hypoxia on fishes: from ecological relevance to physiological effects. Environ. Rev..

[2] Zhang H (2009). Hypoxia-driven changes in the behavior and spatial distribution of pelagic fish and mesozooplankton in the northern Gulf of Mexico. J. Exp. Mar. Biol. Ecol..

[3] Ekau, W. *et al*. *Impacts of hypoxi*a on the structure and processes in pelagic communities (zooplankton, macro-invertebrates and fish). Biogeosciences 7 (2010).

[4] Yu RM (2012). Leptin-mediated modulation of steroidogenic geneexpression in hypoxic zebrafish embryos: implications for the disruption ofsex steroids. Environ. Sci. Technol..

[5] Roberts JJ (2011). Effects of hypoxia on consumption, growth, and RNA: DNA ratios of young yellow perch. Trans. Am. Fish. Soc..

[6] Goodman LR, Campbell JG (2007). Lethal levels of hypoxia for gulf coastestuarine animals. Marine Biol..

[7] Diaz RJ (2001). Overview of hypoxia around the world. J. Environ. Qual..

[8] Geng X (2014). Transcriptional regulation of hypoxia inducible factors alpha (HIF-α) and their inhibiting factor (FIH-1) of channel catfish (*Ictalurus punctatus*) under hypoxia. Comp. Biochem. Physiol. B Biochem. Mol. Biol..

[9] Xiao W (2015). The hypoxia signaling pathway and hypoxic adaptation in fishes. Sci. China. Life. Sci..

[10] Storey KB (1996). Oxidative stress: animal adaptations in nature. Braz. J. Med. Biol. Res..

[11] Li CY, Jackson RM (2002). Reactive species mechanisms of cellular hypoxia- reoxygenation injury. Am. J. Physiol. Cell. Physiol..

[12] Parrilla-Taylar DP, Zenteno-Savín T (2011). Antioxidant enzyme activities in Pacific white shrimp (*Litopenaeus vannamei*) in response to environmental hypoxia and reoxygenation. Aquaculture.

[13] Li SF, Cai WQ, Zhou B (1993). Variation in morphology and biochemical genetic markers among populations of blunt snout bream. Aquaculture.

[14] Zhang D (2001). Study on genetic diversity of bluntnose black bream from Yunihu and Liangzi lakes. J. China. Three. Gorges. Univ..

[15] Xu W, Xiong BX (2008). Advances in the research on genus *Megalobrama* in China. J. Hydroecology..

[16] Ke H (1965). The artificial reproduction and culture experiment of Blunt snout bream. Acta. Hydrobiol. Sin..

[17] Lardon I (2013). 1 H-NMR study of the metabolome of a moderately hypoxia- tolerant fish, the common carp (*Cyprinus carpio*). Metabolomics.

[18] Shen RJ, Jiang XY, Pu JW, Zou SM (2010). HIF-1alpha and -2alpha genes in a hypoxia-sensitive teleost species *Megalobrama amblycephala*: cDNA cloning, expression and different responses to hypoxia. Comp. Biochem. Physiol. B Biochem. Mol. Biol..

[19] Li FG, Chen J, Jiang XY, Zou SM (2015). Transcriptome analysis of blunt snout bream (*Megalobrama amblycephala*) reveals putative differential expression genes related to growth and hypoxia. PLoS One.

[20] Tong C, Zhang CF, Zhang RY, Zhao K (2015). Transcriptome profiling analysis of naked carp (*Gymnocypris przewalskii*) provides insights into the immune-related genes in highland fish. Fish. Shellfish. Immunol..

[21] Qian BY, Xue LY (2016). Liver transcriptome sequencing and de novo annotation of the large yellow croaker (*Larimichthy crocea*) under heat and cold stress. Mar. Genom..

[22] Huang DW, Sherman BT, Lempicki RA (2009). Bioinformatics enrichment tools: paths toward the comprehensive functional analysis of large gene lists. Nucl. Acids Res..

[23] Wang Y (2013). De novo transcriptome sequencing of radish (*Raph anus sativus* L.) andanalysis of major genes involved in glucosinolate metabolism. BMC Genomic.

[24] Hausser J, Zavolan M (2014). Identification and consequences of miRNA-target interactions-beyond repression of gene expression. Nat. Rev. Genet..

[25] Chen PY (2005). Thedevelopmental miRNA profiles of zebrafish as determined by small RNA cloning. Genes Dev..

[26] Daido Y, Hamanishi S, Kusakabe TG (2014). Transcriptional co-regulation of evolutionarily conserved microRNA/cone opsin gene pairs: implications for photoreceptor subtype specification. Dev. Biol..

[27] Lau K (2014). Identification and expression profiling of microRNAs in the brain, liver and gonads of marine medaka (*Oryzias melastigma*) and in response to hypoxia. PLoS One.

[28] Fuller-Carter PI (2015). Integrated analyses of zebrafish miRNA and mRNA expression profiles identify miR-29b and miR-223 as potential regulators of optic nerve regeneration. BMC Genomics.

[29] Wan SM (2016). Dynamic mRNA and miRNA expression analysis in response to intermuscular bone development of blunt snout bream (*Megalobrama amblycephala*). Sci. Rep..

[30] Zhang G (2016). Integrated analysis of mRNA-seq and miRNA-seq in the liver of Pelteobagrus vachelli in response to hypoxia. Sci. Rep..

[31] Tang Z (2015). Integrated analysis of miRNA and mRNA paired expression profiling of prenatal skeletal muscle development in three genotype pigs. Sci. Rep..

[32] Sun SM (2015). Transciptomic and histological analysis of hepatopancreas, muscle and gill tissues of oriental river prawn (*Macrobrachium nipponense*) in response to chronic hypoxia. BMC Genomics.

[33] Gracey AY, Troll JV, Somero GN (2001). Hypoxia-induced gene expression profiling in the euryoxic fish *Gillichthys mirabilis*. Proc. Natl. Acad. Sci..

[34] Sollid J, DeAngelis P, Gundersen K, Nilsson GE (2003). Hypoxia induces adaptive and reversible grossmorphological changes in crucian carp gills. J. Exp. Biol..

[35] Tiedke J, Thiel R, Burmester T (2014). Molecular response of estuarine fish to hypoxia: a comparative study with ruffe and flounder from field and laboratory. PLoS One.

[36] Hall JR (2009). Expression levels of genes associated with oxygen utilization, glucose transport and glucose phosphorylation in hypoxia exposed Atlantic cod (*Gadus morhua*). Comp. Biochem. Physiol. D Genomics Proteomics..

[37] Chen BX (2017). Transcriptome comparison reveals insights into muscle response to hypoxia in blunt snout bream (*Megalobrama amblycephala*). Gene.

[38] Chandel NS (1998). Mitochondrial reactive oxygen species trigger hypoxia-induced transcription. Proc. Natl. Acad. Sci. USA.

[39] Chandel NS (2000). Reactive oxygen species generated atmitochondrial complex III stabilize hypoxia-inducible factor1-α during hypoxia. J. Biol. Chem..

[40] Turrens JF (2003). Mitochondrial formation of reactive oxygen species. J. Physiol. London..

[41] Dolci GS (2017). Could hypoxia acclimation cause morphological changes and protect against Mn-induced oxidative injuries in silver catfish (*Rhamdia quelen*) even after reoxygenation?. Environ. Pollut..

[42] Lushchak VI, Bagnyukova TV, Lushchak OV, Storey JM, Storey KB (2005). Hypoxia and recovery perturb free radical processes and antioxidant potential in common carp (*Cyprinus carpio*) tissues. Int. J. Biochem. Cell Biol..

[43] Zhang GS (2016). Modulated expression and enzymatic activities of Darkbarbel catfish, *Pelteobagrus vachelli* for oxidative stress induced by acute hypoxia and reoxygenation. Chemosphere.

[44] Chen N (2017). Effects of acute hypoxia and reoxygenation on physiological and rmmune responses and redox balance of Wuchang bream (*Megalobrama amblycephala* Yih, 1955). Front. Physiol..

[45] Mao X, Cai T, Olyarchuk JG, Wei L (2005). Automated genome annotation and pathway identification using the KEGG Orthology (KO) as a controlled vocabulary. Bioinformatics..

[46] Fukushima A, Kusano M, Redestig H, Arita M, Saito K (2011). Metabolomic correlation network modules in Arabidopsis based on a graph-clustering approach. BMC Syst Biol.

[47] Semenza GL (2000). HIF-1: mediator of physiological and pathophysiological responses to hypoxia. J. Appl. Physiol..

[48] Semenza GL (2003). Targeting HIF-1 for cancer therapy. Nat. Rev. Cancer.

[49] Wenger RH (2000). Mammalian oxygen sensing, signalling and gene regulation. J. Exp. Biol..

[50] Fandrey J, Gorr TA, Gassmann M (2006). Regulating cellular oxygen sensing by hydroxylation. Cardiovasc. Res..

[51] Nikinmaa, M. & Rees, B. B. Oxygen-dependent gene expression in fishes. *Am. J. Physiol. - Reg. I*. **288**, R1079–R1090 (2005). 10.1152/ajpregu.00626.2004 15821280

[52] Jibb LA, Richards JG (2008). AMP-activated protein kinase activity during metabolic rate depression in the hypoxic goldfish, *Carassius auratus*. J. Exp. Biol..

[53] Ellefsen SO, Stecyk JAW (2008). Differential regulation of AMP-activated kinase and AKT kinase in response to oxygen availability in crucian carp (*Carassius carassius*). Am. J. Physiol.: Regul. Integr. Comp. Physiol..

[54] Xiao WH (2015). The hypoxia signaling pathway and hypoxic adaptation in fishes. Sci. China Life. Sci..

[55] Fan XY (2015). Activation of the AMPK-ULK1 pathway plays an important role in autophagy during prion infection. Sci. Rep..

[56] Chen N (2012). Molecular characterization and expression analysis of three hypoxia-inducible factor alpha subunits, HIF-1α/2α/3α of the hypoxia-sensitive freshwater species, Chinese sucker. Gene.

[57] Pichavant K (2002). Effects of hypoxia and subsequent recovery on turbot *Scophtalmus maximus*: hormonal changes and anaerobic metabolism. Mar. Ecol. Prog. Ser..

[58] Chabot D, Claireaux G (2008). Environmental hypoxia as a metabolic constraint on fish: the case of Atlantic cod. Gadus morhua. Mar. Pollut. Bull..

[59] Routley MH, Nilsson CE, Renshaw GMC (2002). Exposure to hypoxia primers the respiratory and metabolic responses of the epaulette shark to progressive hypoxia. Comp. Biochem. Physiol. A Mol. Integr. Physiol..

[60] Li XF, Liu WB, Lu KL, Xu WN, Wang Y (2012). Dietary carbohydrate/lipid ratios affect stress, oxidative status and non-specific immune responses of fingerling blunt snout bream, *Megalobrama amblycephala*. Fish. Shellfish Immunol..

[61] Delaloy C (2010). MicroRNA-9 coordinates proliferation and migration of human embryonic stem cell–derived neural progenitors. Cell. Stem. Cell..

[62] Gao FB (2010). Context-dependent functions of specific microRNAs in neuronal development. Neural Dev.

[63] Kawahara H, Imai T, Okano H (2012). MicroRNAs in neural stem cells and neurogenesis. Front. Neurosci..

[64] Yao M (2014). Dicer mediating the expression of miR-143 and miR-155 regulates hexokinase II associated cellular response to hypoxia. Am. J. Physiol.: Lung Cell. Mol. Physiol..

[65] Xu X, Liu C, Bao J (2017). Hypoxia-induced hsa-miR-101 promotes glycolysis by targeting TIGAR mRNA in clear cell renal cell carcinoma. Mol Med Rep..

[66] Tsai HJ, Wilson JE (1997). Functional organization of mammalian hexokinases: Characterization of the rat type III isozyme and its chimeric forms, constructed with the N- and C-terminal halves of the type I and type II isozymes. Arch. Biochem. Biophys..

[67] Yan HL (2009). Repression of the miR-17-92 cluster by p53 has an important function in hypoxia-induced apoptosis. EMBO. J..

[68] Nallamshetty S, Chan SY, Loscalzo J (2013). Hypoxia: a master regulator of microRNA biogenesis and activity. Free. Radic. Biol. Med..

[69] Chan JA, Krichevsky AM, Kosik KS (2005). MicroRNA-21 is an anti-apoptotic factor in human glioblastoma cells. Cancer Res.

[70] Xiong Y (2010). Effects of microRNA-29 on apoptosis, tumorigenicity, and prognosis of hepatocellular carcinoma. Hepatology.

[71] Koumenis C (2001). Regulation of p53 by hypoxia: dissociation of transcriptional repression and apoptosis from p53-dependent transactivation. Mol. Cell. Biol..

[72] Sun SM, Zhu J, Jiang XJ, Li B, Ge XP (2014). Molecular cloning, tissue distribution and expression analysis of a manganese superoxide dismutase in blunt snout bream *Megalobrama amblycephala*. Fish. Shellfish. Immunol..

[73] Pei H (2013). Integrative analysis of miRNA and mRNA profiles in response to ethylene in rose petals during flower opening. PloS One.

[74] Sun SM (2017). Molecular cloning, characterization and expression analysis of caspase-3 from the oriental river prawn, *Macrobrachium nipponense* when exposed to acute hypoxia and reoxygenation. Fish. Shellfish. Immunol..

[75] Grabherr MG (2011). Full-length transcriptome assembly from RNA-Seq data without a reference genome. Nat. Biotechnol..

[76] Koonin EV (2004). Acomprehensive evolutionary classification of proteins encoded in complete eukaryotic genomes. Genome Biol..

[77] Boeckmann B (2003). *The SWISS-PROT protei*n knowledge base and its supplement TrEMBL in 2003. Nucl. Acids Res..

[78] Kanehisa M (2008). KEGG for linking genomes to life and the environment. Nucleic Acids Res..

[79] Kanehisa M, Sato Y, Kawashima M, Furumichi M, Tanabe M (2016). KEGG as a reference resource for gene and protein annotation. Nucleic Acids Res..

[80] Marioni JC, Mason CE, Mane SM, Stephens M, Gilad Y (2008). RNA-seq an assessment of technical reproducibility and comparison with gene expression arrays. Genome Res..

[81] Robinson MD, McCarthy DJ, Smyth G (2010). K. edgeR: a Bioconductor package for differential expression analysis of digital gene expression data. Bioinformatics..

[82] Sun SM, Ge XP, Zhu J, Zhang WX, Xuan FJ (2016). De novo assembly of the blunt snout bream (*Megalobrama amblycephala*) gill transcriptome to identify ammonia exposure associated microRNAs and their targets. Results. Immunol..

[83] Wei M (2014). Identification and profiling of sex-biased microRNAs from sea urchin Strongylocentrotus nudus gonad by Solexa deep sequencing. Com. Biochem. Phys. D Genomics. Proteomics..

[84] Schmittgen L (2001). Analysis of relative gene expression data using real-time quantitative PCR and the 2^−ΔΔCT^ method. Methods.

