# Supplementary material for: Dynamic mRNA and miRNA expression analysis in response to hypoxia and reoxygenation in the blunt snout bream (Megalobrama amblycephala)
Source: Sci Rep. 2017 Oct 9;7:12846. doi: 10.1038/s41598-017-12537-7 (PMC5634510; doi:10.1038/s41598-017-12537-7)

**Dynamic mRNA and miRNA expression analysis in response to hypoixa and reoxygenation of blunt snout bream (*Megalobrama amblycephala*)**

Shengming Sun1, Fujun Xuan2, Xianping Ge1*, Jian Zhu1*, Wuxiao Zhang3

1 Key Laboratory of Genetic Breeding and Aquaculture Biology of Freshwater Fishes, Ministry of Agriculture, Freshwater Fisheries Research Centre, Chinese Academy of Fishery Sciences, Wuxi 214081, PR China

2 Jiangsu Provincial Key Laboratory of Coastal Wetland Bioresources and Environmental Protection, Yancheng City, Jiangsu Province 224002, PR China

3 Wuxi Fisheries College, Nanjing Agricultural University, Wuxi 214081, PR China

*Corresponding authors: Xianping Ge, Jian Zhu

Freshwater Fisheries Research Center, Chinese Academy of Fishery Sciences, No.9 East Shanshui Road. Wuxi, Jiangsu 214081, P. R. China

E-mail: [gexp@ffrc.cn](mailto:gexp@ffrc.cn), [zhuj@ffrc.cn](mailto:zhuj@ffrc.cn). Tel.: +86 510 85557892

**Keywords:** blunt snout bream; hypoxia; RNA-seq; miRNA-mRNA interaction; liver

Figure S1 Length distribution of contigs in the transcriptome

Figure S2 The results of NR annotation. (A): The species distribution of the result of NR annotation. (B): The E-value distribution of the result of NR annotation. (C): The similarity distribution of the result of NR annotation.

Figure S3 Principal component analysis (PCA) of 12 DGE libraries in the 4 stages. The lines of different colors represent different DGE libraries. The length of the lines and the distance between lines represents the difference between each other.

Figure S4 KEGG pathway enrichment analyses (20 pathways). (A): Pathway enrichment for hypoxia 3h vs. normoxia. (B): Pathway enrichment for hypoxia 24h vs. normoxia. (C): Pathway enrichment for reoxygenation 3h vs. normoxia.

Figure S5. Significantly differentiated expressed genes that were identified by KEGG as involved in HIF-1 pathway in hypoxia 3 h vs. normoxia. Red boxes indicate significantly increased expression. Black boxes indicate unchanged expression.

Kanehisa, M., Furumichi, M., Tanabe, M., Sato, Y., Morishima, K. KEGG: new perspectives on genomes, pathways, diseases and drugs. *Nucleic Acids Res*. **45**, D353-D361 (2017).

Kanehisa, M., Sato, Y., Kawashima, M., Furumichi, M., Tanabe, M. KEGG as a reference resource for gene and protein annotation. *Nucleic Acids Res*. **44**, D457-D462 (2016).

Kanehisa, M., Goto, S. KEGG: Kyoto Encyclopedia of Genes and Genomes. *Nucleic Acids Res*. **28(1)**, 27-30 (2000).

Figure S1


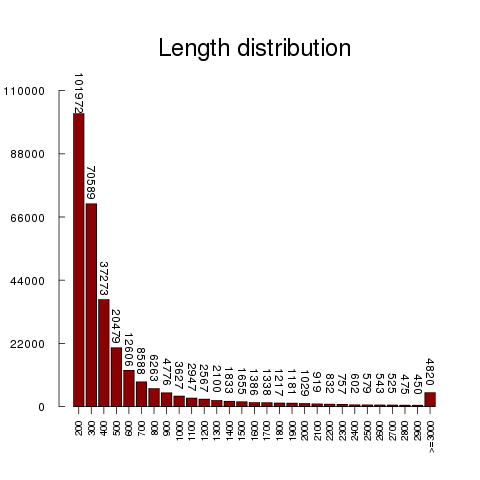


Figure S2


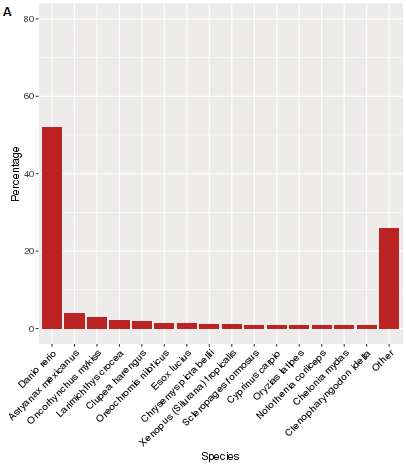


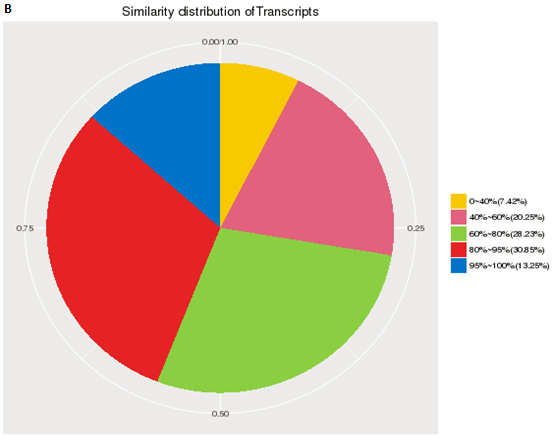


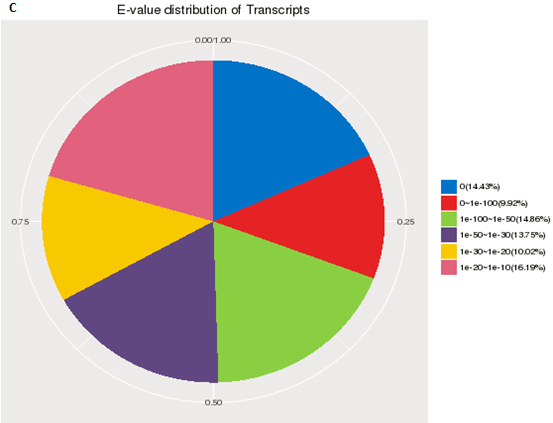


Figure S3


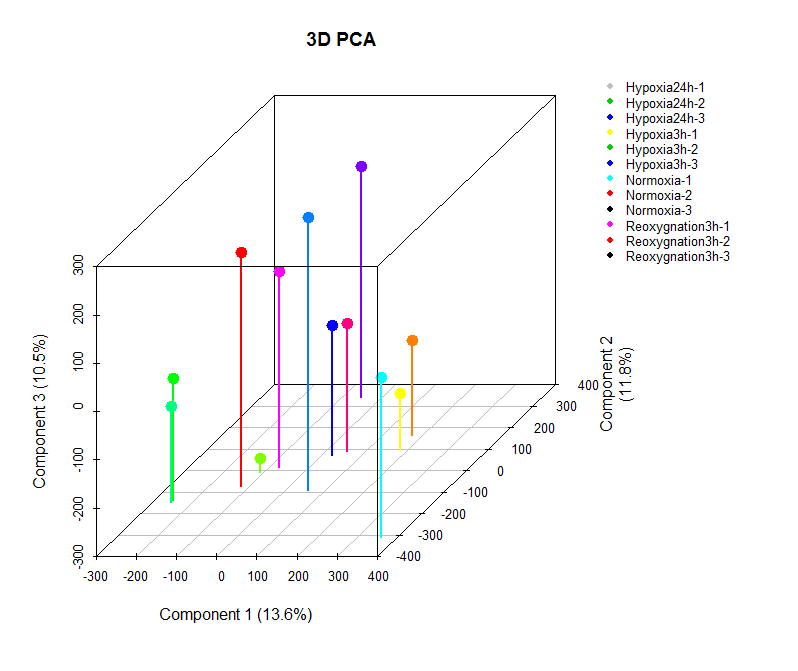


Figure S4


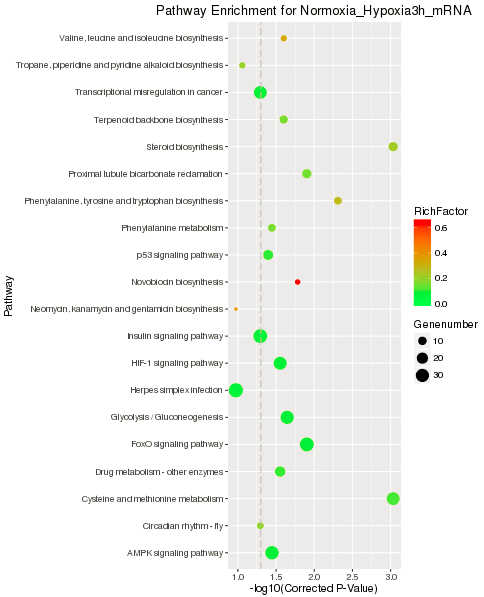


**A**


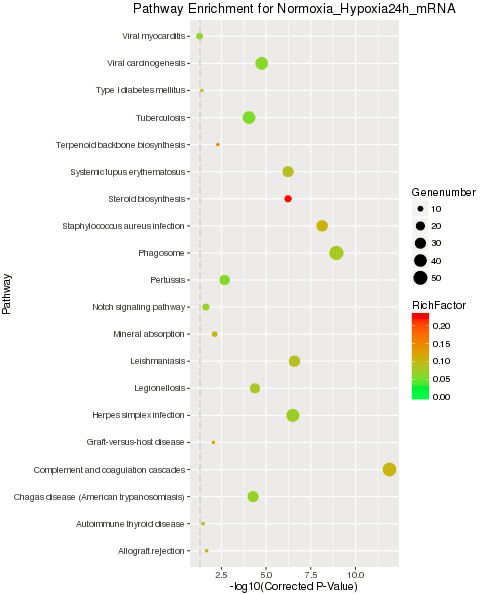


**B**


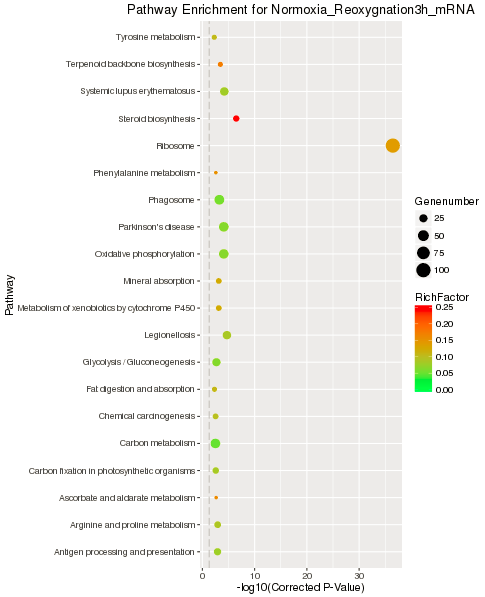


**C**

Figure S5


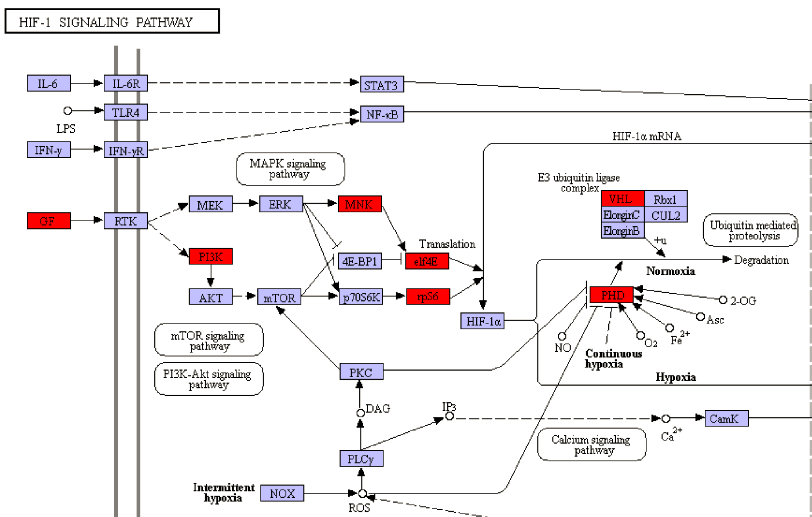

Supplement: Supplementary file 1 — Additional figures [file 41598_2017_12537_MOESM1_ESM.doc]
